# Dopamine-Induced Changes in Gα_olf_ Protein Levels in Striatonigral and Striatopallidal Medium Spiny Neurons Underlie the Genesis of l-DOPA-Induced Dyskinesia in Parkinsonian Mice

**DOI:** 10.3389/fncel.2017.00026

**Published:** 2017-02-10

**Authors:** Ryoma Morigaki, Shinya Okita, Satoshi Goto

**Affiliations:** ^1^Department of Neurodegenerative Disorders Research, Institute of Biomedical Sciences, Graduate School of Medical Sciences, Tokushima UniversityTokushima, Japan; ^2^Parkinson’s Disease and Dystonia Research Center, Tokushima University HospitalTokushima, Japan; ^3^Department of Neurosurgery, Institute of Biomedical Sciences, Graduate School of Medical Sciences, Tokushima UniversityTokushima, Japan

**Keywords:** olfactory type G-protein α subunit, dopamine, striatum, Parkinson’s disease, L-DOPA-induced dyskinesia

## Abstract

The dopamine precursor, l-3,4-dihydroxyphenylalanine (l-DOPA), exerts powerful therapeutic effects but eventually generates l-DOPA-induced dyskinesia (LID) in patients with Parkinson’s disease (PD). LID has a close link with deregulation of striatal dopamine/cAMP signaling, which is integrated by medium spiny neurons (MSNs). Olfactory type G-protein α subunit (Gα_olf_), a stimulatory GTP-binding protein encoded by the *GNAL* gene, is highly concentrated in the striatum, where it positively couples with dopamine D_1_ (D_1_R) receptor and adenosine A_2A_ receptor (A_2A_R) to increase intracellular cAMP levels in MSNs. In the striatum, D_1_Rs are mainly expressed in the MSNs that form the striatonigral pathway, while D_2_Rs and A_2A_Rs are expressed in the MSNs that form the striatopallidal pathway. Here, we examined the association between striatal Gα_olf_ protein levels and the development of LID. We used a hemi-parkinsonian mouse model with nigrostriatal lesions induced by 6-hydroxydopamine (6-OHDA). Using quantitative immunohistochemistry (IHC) and a dual-antigen recognition *in situ* proximity ligation assay (PLA), we here found that in the dopamine-depleted striatum, there appeared increased and decreased levels of Gα_olf_ protein in striatonigral and striatopallidal MSNs, respectively, after a daily pulsatile administration of l-DOPA. This leads to increased responsiveness to dopamine stimulation in both striatonigral and striatopallidal MSNs. Because Gα_olf_ protein levels serve as a determinant of cAMP signal-dependent activity in striatal MSNs, we suggest that l-DOPA-induced changes in striatal Gα_olf_ levels in the dopamine-depleted striatum could be a key event in generating LID.

## Introduction

Human pathology has shown that Parkinson’s disease (PD) results from dopamine deficiency in the neostriatum, particularly in the putamen, due to degenerative loss of nigrostriatal dopaminergic cells (Kish et al., 1988; Goto et al., 1989). Treatments with the dopamine precursor, l-3,4-dihydroxyphenylalanine (l-DOPA), remain the gold standard of drug therapy for PD. However, after *prolonged* and *pulsatile* exposure to L-DOPA, PD patients eventually develop L-DOPA-induced dyskinesia (LID; Jenner, 2008; Calabresi et al., 2010; Huot et al., 2013). LID is an adverse event that occurs in more than 50% of patients after 5–10 years (Ahlskog and Muenter, 2001; Rascol et al., 2006). Importantly, once LID has been established, its severity increases unless dopaminergic drug dosage is reduced (Brotchie, 2005). It is known that the severity of loss of nigral dopaminergic cells represents the most important factor that determines the severity of LID (Guridi et al., 2012; Bastide et al., 2015). However, the nature of the cellular and molecular key events that lead to a progressive increase in responsiveness to dopaminergic stimulation in LID remains unclear.

LID is closely linked with pathological changes in dopaminergic transmissions in the striatum (Bastide et al., 2015; Calabresi et al., 2016). Dopamine receptors are categorized into two subclasses, D_1_- and D_2_-type receptors, based on their functional properties to stimulate and inhibit the adenylyl cyclase-mediated cAMP production via specific targeting of G-proteins, respectively (Kebabian and Calne, 1979; Missale et al., 1998). There is a large body of evidence showing that increased activity of dopamine D_1_-receptors (D_1_Rs) is necessary for LID development (Westin et al., 2007; Darmopil et al., 2009; Alcacer et al., 2012). D_1_R activation leads to multiple molecular events, such as the induction of immediate early genes (Cenci et al., 1999; Gerfen et al., 2002; Darmopil et al., 2009) and the activation of extracellular signal-regulated kinases (Gerfen et al., 2002; Pavón et al., 2006; Santini et al., 2007, 2009; Westin et al., 2007; Rylander et al., 2009; Ding et al., 2011). Striatal dopamine/cAMP signaling is integrated by medium spiny neurons (MSNs), which are the principal neurons of the striatum (Graybiel, 2008; Kreitzer, 2009; Gerfen and Surmeier, 2011). MSNs can be divided into two distinct subpopulations on the basis of their axon projections, which form the “direct” striatonigral and “indirect” striatopallidal pathways (Crittenden and Graybiel, 2011; Gerfen and Surmeier, 2011). Interestingly, anatomical evidence has shown that D_1_Rs and D_2_Rs are mainly expressed in striatonigral and striatopallidal MSNs, respectively. Moreover, adenosine A_2A_ receptor (A_2A_R), a prototypical Gs-coupled receptor, is enriched in the striatum, where it is mainly expressed in striatopallidal, but not striatonigral, MSNs (Schiffmann et al., 1991; Svenningsson et al., 1999; Schwarzschild et al., 2006; Fuxe et al., 2007).

Olfactory type G-protein α subunit (Gα_olf_), the stimulatory G-protein encoded by the *GNAL* gene, is highly concentrated in the striatum, where it positively couples with D_1_R and A_2A_R to activate adenylyl cyclase and, thereby, increase intracellular cAMP levels in MSNs (Hervé, 2011). As Gα_olf_ represents the rate-limiting factor for the D_1_R- and A_2A_R-dependent cAMP production (Kull et al., 2000; Corvol et al., 2001), Gα_olf_ protein level serves as a determinant of cAMP signal-dependent activity in both D_1_R-expressing striatonigral MSNs (D1-cells) and D_2_R-expressing striatopallidal MSNs (D2-cells). D_1_R/Gα_olf_-mediated increases in intracellular cAMP levels facilitate D1-cell activity (Hervé, 2011), while the elevation of intracellular cAMP levels via A_2A_R/Gα_olf_ activation functionally opposes the actions of D_2_Rs on D2-cells (Schwarzschild et al., 2006; Fuxe et al., 2007). It is also known that Gα_olf_ protein levels in striatal MSNs are regulated by posttranslational usage-dependent mechanism through the activation of D_1_Rs (Hervé et al., 2001; Corvol et al., 2004, 2007; Alcacer et al., 2012; Ruiz-DeDiego et al., 2015) and A_2A_Rs (Hervé et al., 2001).

The aim of this study was to clarify the association of striatal Gα_olf_ protein levels with LID development. For this purpose, we used a hemi-parkinsonian mouse model with nigrostriatal lesion induced by 6-hydroxydopamine (6-OHDA). Using quantitative immunohistochemistry (IHC) and a highly-sensitive *in situ* proximity ligation assay (PLA), we show that in the 6-OHDA-lesioned striatum, daily *pulsatile* injections of L-DOPA might cause changes in Gα_olf_ levels in not only D1-cells but also D2-cells, and lead to elevated responsiveness to dopamine stimulation in both D1-cells and D2-cells. This novel finding suggests that L-DOPA-induced changes in striatal Gα_olf_ levels in the dopamine-denervated striatum may serve as a principal cause for generating LID.

## Materials and Methods

### Experimental Animals

All experimental procedures involving the use of animals and the analysis of brain anatomy were approved by the Institutional Care and Use Committees of Tokushima University, Japan. Adult male C57BL/6 mice aged 8–9 weeks were purchased from Nihon SLC Co. (Shizuoka, Japan). Mice were housed in a controlled environment (23 ± 1°C, 50 ± 5% of humidity) with 12 h light/dark cycle. Mice were allowed to take food and tap water *ad libitum*.

### Stereotaxic Injection of 6-OHDA

Mice were anesthetized with isoflurane (Sigma-Aldrich, St. Louis, MO, USA) and were mounted on a stereotaxic frame (Narishige, Tokyo, Japan). Each mouse received a stereotaxic injection of 6-OHDA-HCl (8.2 μg) dissolved in 4 μl of saline containing 0.02% ascorbic acid. Two 2-μl injections were administered into the striatum at a rate of 1 μl/min. The needle was left in place for 5 min to allow diffusion away from the injection site. The stereotaxic coordinates according to the mouse brain atlas (Paxinos and Franklin, 2001) were anterior-posterior, +0.5; medial-lateral, +2.4; and dorsal-ventral, −4.0 and −3.0. Mice were allowed to recover for 3 weeks and then apomorphine (Sigma-Aldrich; 0.5 mg/kg)-induced rotation behavior was studied over the course of 60 min. Mice with contralateral rotations (>7 times/min) were chosen and used for further studies.

### l-DOPA Treatments

Three weeks after the 6-OHDA-lesioning, mice received intraperitoneal injections of L-DOPA (Sigma-Aldrich; 20 mg/kg of free base) dissolved in 0.9% saline and intraperitoneal injections of benserazide-HCl (Sigma-Aldrich; 12 mg/kg) dissolved in 0.9% saline 20 min before daily administration of L-DOPA over 10 days. On day 11, the mice underwent behavioral studies and were then sacrificed for histological studies.

### Assessment of Abnormal Involuntary Movements (AIMs)

AIM scoring was performed according to previous reports (Cenci et al., 1998; Pavón et al., 2006; Santini et al., 2007). AIM scores were obtained after the last injection of L-DOPA for 1 min every 10 min over a period of 140 min. For the evaluation, each mouse was placed in a glass cylinder (diameter of 12 cm). Purposeless movements were classified on the basis of their topographic distribution. The following four subtypes of AIMs were present: locomotive (tight contralateral turns), axial (twisted posturing of the neck and upper body toward the contralateral side), forelimb (jerky movements of the contralateral forelimb, and/or grabbing movement of the contralateral paw), and orolingual (jaw movements and tongue protrusion toward the contralateral side). Each subtype was scored as follows; 0, absent; 1, occasional; 2, frequent; 3, continuous; 4, continuous, not interrupted by sensory stimuli.

### Tissue Preparations

Immediately after the last AIM scoring, the mice were intraperitoneally administered a lethal dose of pentobarbital (Sigma-Aldrich). They were then transcardially perfused with 0.01 M phosphate-buffered saline (PBS) at pH 7.2, followed by cold 4% paraformaldehyde in 0.1 M phosphate buffer at pH 7.2. The brains were removed, post-fixed overnight in the same fixative at 4°C, and stored in a 10–30% sucrose gradient in 0.1 M phosphate buffer at 4°C for cryoprotection. Sixteen-micrometre-thick sections were cut on a cryostat and stored in PBS containing 0.05% NaN_3_ until use.

### IHC

Immunostaining was performed on free-floating sections using the tyramide signal amplification (TSA) method, as in our previous report (Okita et al., 2012). After blocking endogenous peroxidase activity, the sections were incubated in PBS containing 3% bovine serum albumin (BSA) for 60 min. They were then incubated with antibodies against one of the following (diluted in PBS-BSA): Gα_olf_ (rabbit polyclonal, 1:5000; Santa Cruz Biotechnology, Santa Cruz, CA, USA), tyrosine hydroxylase (TH, rabbit polyclonal, 1:100,000) (Sato et al., 2008; Morigaki and Goto, 2016), D_1_R (mouse monoclonal, 1:5000; Novus Biologicals, Littleton, CO, USA), A_2A_R (mouse monoclonal, 1:5000; Santa Cruz Biotechnology), D_2_R (rabbit polyclonal, 1:2000; Merck Millipore, Billerica, MA, USA) or c-Fos (rabbit polyclonal, 1:50,000; Oncogene Science, Cambridge, MA, USA) for 18 h. The bound antibodies were detected using the Histofine Simple Stain Kit (Nichirei, Tokyo, Japan) and the TSA-system with Cyanine3 or Fluorescein (Perkin Elmer, Shelton, CT, USA). For double immunofluorescence staining, the sections stained for Gα_olf_ using Cyanine3 were incubated in 0.1 M glycine-HCl (pH 2.2) at room temperature for 30 min. After rinsing in PBS for 1 h, the sections were then incubated overnight at room temperature in PBS containing 3% BSA and a rabbit polyclonal antibody against the μ-opioid receptor (MOR; 1:20,000; Millipore, Billerica, MA, USA), a mouse monoclonal antibody against D_1_R (1:5000; Novus Biologicals), or a mouse monoclonal antibody against A_2A_R (1:5000; Santa Cruz Biotechnology). The bound antibodies were detected using the Histofine Simple Stain Kit (Nichirei) and the TSA-system with Fluorescein (Perkin Elmer).

### Dual-Antigen Recognition *In Situ* PLA

Dual-antigen recognition PLA experiments were conducted using the *Brightfield* Duolink PLA kit reagents (Sigma-Aldrich) according to the manufacturer’s recommendations with some modifications. Briefly, after blocking endogenous peroxidases in PBS containing 0.1% H_2_O_2_ for 30 min, the free-floating sections were incubated in PBS containing 3% normal goat serum for 60 min. They were then incubated in PBS containing 3% normal goat serum and a rabbit polyclonal antibody against Gα_olf_ (1:500; Santa Cruz Biotechnology) in combination with a mouse monoclonal antibody against D_1_R (1:500; Novus Biologicals) or a mouse monoclonal antibody against A_2A_R (1:500; Santa Cruz Biotechnology) for 18 h at room temperature. After subsequent secondary labeling with rabbit PLA *minus* and mouse PLA *plus* probes, we used the Brightfield Duolink Detection reagents for ligation and amplification and label probe binding according to the manufacturer’s instructions. For final signal visualization, we used the TSA-system with Cyanine3 (Perkin Elmer). After mounting on slides, the stained sections were counterstained with hematoxylin and were cover-slipped using 10% glycerol in PBS.

### Digital Imaging and Morphometry

Digital microscopy images were captured using an Olympus BX51 microscope (Olympus, Tokyo, Japan) equipped with a DP40 digital camera (Olympus). They were imported into Adobe Photoshop CS4 and processed digitally. We adjusted contrast, brightness, and color balance. Using an image analyzer (MetaMorph, Molecular Device, Tokyo, Japan), we measured the optical densities of immunoreactive products and PLA signals in the striatum, which were represented by gray levels on non-colored digital images (Sato et al., 2008; Goto et al., 2013; Morigaki and Goto, 2015). Using the same protocol described above, we also measured optical densities of Gα_olf_-immunoreactive products in the striosome and matrix subfields in the striatal sections double-stained for Gα_olf_ and MOR. We also counted the numbers of neuronal nuclei positive for c-Fos in a 0.5 mm × 0.5 mm field in the striatum and globus pallidus, as in our previous report (Tanabe et al., 2014). These morphometric analyses were carried out in a blind manner.

### Statistical Analysis

All experimental values are expressed as means ± SEM. For two-group comparisons, we used a paired two-tailed *t*-test. Multiple comparisons were analyzed using one-way or two-way analysis of variance (ANOVA), followed by Bonferroni’s *post hoc* tests for pair wise comparisons. Statistical analyses were performed using Stat View 5.0 (SAS Institute, Cary, NC, USA) software. *P*-values of less than 0.05 were considered statistically significant.

## Results

### Generation of a Mouse Model with LID

**Figure 1:**
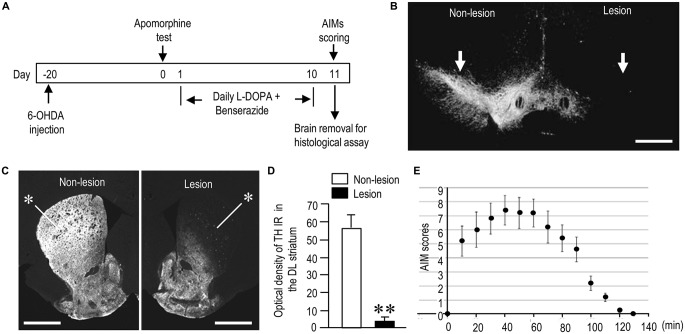
**Generation of hemi-parkinsonian mice with l-DOPA-induced dyskinesia (LID). (A)** Timeline of treatments and observations (also see “Materials and Methods” Section). **(B,C)** Representative photomicrographs of the substantia nigra (**B**, arrows) and striatum (**C**, asterisks) stained for tyrosine hydroxylase (TH) on the non-lesioned (Non-lesion) and lesioned (Lesion) sides in mice with 6-hydroxydopamine (6-OHDA)-induced lesions. **(D)** Quantification of mean TH staining intensity in the dorsolateral (DL) striatum on the non-lesioned (Non-lesion) and lesioned (Lesion) sides. Data are means ± SEM (*n* = 10). Paired two-tailed *t* test: **P < 0.01 vs. Non-lesion. **(E)** Time course of total abnormal involuntary movements (AIMs) scored every 10 min over a period of 140 min after the last L-DOPA administration. Data are means ± SEM at the each time point (*n* = 10 per group). Scale bars: **(B)** = 1 mm; **(C)** = 2 mm.

To model the generation of AIMs in PD following repeated L-DOPA treatments, we employed a well-established PD mouse model in which mice first received unilateral injection of 6-OHDA into the striatum (Santini et al., 2007). After 3 weeks of recovery and an apomorphine test, the mice were subjected to L-DOPA treatment for 10 days according to standardized protocols (Figure 1A). In this study, 6-OHDA-lesioned mice administered daily injections of benserazide-HCl (12 mg/kg) alone for 10 days were designated as “PD” models. Six-OHDA-lesioned mice that received daily injections of L-DOPA (20 mg/kg) and benserazide-HCl (12 mg/kg) for 10 days and finally exhibited LIDs with total AIM scores of more than 20 were designated as “PD with Dyskinesia (PD-D)” models. Among 6-OHDA-lesioned mice that received daily injections of L-DOPA (*n* = 28), 25 mice (~90%) were grouped into the PD-D model. Mice that received no drug treatment, except for anesthetic drugs, were used as “naïve controls”.

In both PD and PD-D mice, IHC with anti-TH antibody revealed a severe loss of nigral dopaminergic cells (Figure 1B) and striatal dopaminergic afferents (Figure 1C) on the side of the 6-OHDA injection. Quantitative measurements (Figure 1D) revealed a greater-than-90% reduction in TH labeling in the dorsolateral (DL) striatum on the lesioned side when compared to the non-lesioned side (lesion side, 3.9 ± 2.1; non-lesion side, 56.5 ± 8.9; means ± SEM; *n* = 10; two-tailed *t*-test, *P* < 0.01). Figure 1E shows the time course of changes in LIDs as determined by AIM scoring in PD-D mice. AIMs were maximal 40 min after L-DOPA administration, declined after 70 min, and almost disappeared after 120 min.

### Regional and Cellular Localization of Gα_olf_ in the Normal Mouse Striatum

**Figure 2:**
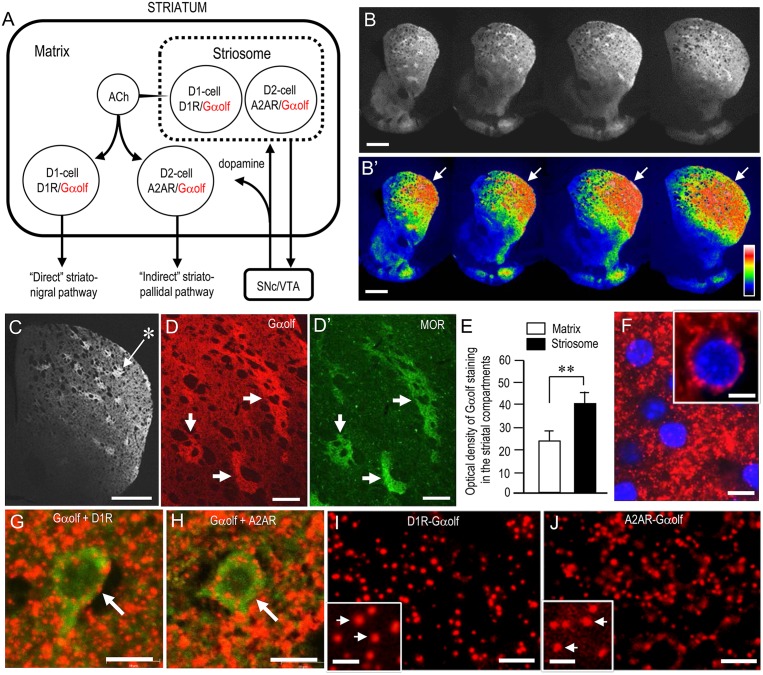
**Striatal localization of Gα_olf_ protein in normal mice. (A)** Localization pattern of Gα_olf_ in a simplified basal ganglia circuit. Note that Gα_olf_ is colocalized with D_1_R in striatonigral medium spiny neurons (MSNs; D1-cells), but with adenosine A2A receptor (A2AR) in D_2_R-expressing striatopallidal MSNs (D2-cells). The striatonigral and striatopallidal pathways arising from the striosome are omitted in this scheme. Abbreviations: SNc, substantia nigra pars compacta; VTA, ventral tegmental area. **(B,B’)** Multiple frontal sections of the striatum stained for Gα_olf_ from naïve control mice **(B)** and their graded color-converted images **(B’)**. Note that Gα_olf_ immunoreactivity (IR) is highly concentrated in the DL portion of the striatum (arrows). **(C)** Representative photomicrograph of the striatum stained for Gα_olf_. Asterisk indicates an example of the striosomes. **(D,D’)** Representative photomicrographs of the DL striatum double-stained for Gα_olf_ **(D)** and μ-opioid receptor (MOR) **(D’)**. Corresponding striosomes are indicated by arrows. **(E)** Quantification of mean Gα_olf_ staining intensity in the striosome and matrix compartments in the DL striatum. Data are means ± SEM (*n* = 10). Paired two-tailed Student’s *t* test: **P < 0.01, Striosome vs. Matrix. **(F)** Representative photomicrographs of the DL striatum stained for Gα_olf_ with DAPI (4,6-diamidino-2-phenylindole)-staining. Tiny dots positive for Gα_olf_ (*inset*) are shown. **(G,H)** Representative photomicrographs of neurons double-stained for Gα_olf_ and D_1_R **(G)** or A_2A_R **(H)** in the DL striatum. **(I,J)** Representative photomicrographs of the DL striatum stained with the dual recognition *in situ* proximity ligation assay (PLA) for Gα_olf_-D_1_R **(I)** or Gα_olf_-A_2A_R **(J)**. Tiny dots showing the PLA signals for Gα_olf_-D_1_R **(I)** or Gα_olf_-A_2A_R **(J)** are abundant. Microscopic images at higher magnifications are shown in the *insets* (arrows) in **(I,J)**. Scale bars: **(B,C)** = 1 mm; **(D,D’)** = 200 μm: **(F–J)** = 10 μm; *inset* in **(F)** = 5 μm; *insets* in **(I,J)** = 2.5 μm.

Figure 2A depicts the known distributional patterns of Gα_olf_, D_1_R, and A_2A_R in a simplified basal ganglia circuit diagram. Note that Gα_olf_ is mainly localized with D_1_R in the D1-cells that form the striatonigral pathway, while it is localized with A_2A_R in the D2-cells that form the striatopallidal pathway. Using IHC, we reappraized the localization profile of Gα_olf_ immunoreactivity (IR) in the mouse striatum. Low-magnification microscopic images show strong Gα_olf_ labeling in the striatum (Figure 2B), particularly in the DL region (Figure 2B’, arrows). As in our previous report (Sako et al., 2010), Gα_olf_ IR was differentially concentrated in the different striatal compartments, with heightened Gα_olf_ labeling in the striosomes relative to the matrix (Figures 2C,D). Optical density measurements (Figure 2E) also revealed that Gα_olf_ IR in the striosomes was significantly higher than that in the matrix (striosomes, 39.8 ± 5.0; matrix, 23.5 ± 6.0; means ± SEM; *n* = 10; two-tailed *t*-test, *P* < 0.01). Microscopic images with high magnification show numerous tiny dots of Gα_olf_ IR densely distributed in the DL striatum (Figures 2F–H). In the double-labeling study, Gα_olf_-positive dots were frequently localized in MSNs labeled for D_1_R (Figure 2G) or A_2A_R (Figure 2H). Using dual-antigen recognition *in situ* PLA, which indicates that two proteins are in close proximity (Söderberg et al., 2006), we also found that dot signals indicating the presence of Gα_olf_ protein in close proximity to D_1_R protein (D_1_R-Gα_olf_; Figure 2I) or A_2A_R protein (A_2A_R-Gα_olf_; Figure 2J) were abundantly distributed in the DL striatum.

### Dopaminergic Regulation of Striatal Gα_olf_ Protein Levels

**Figure 3:**
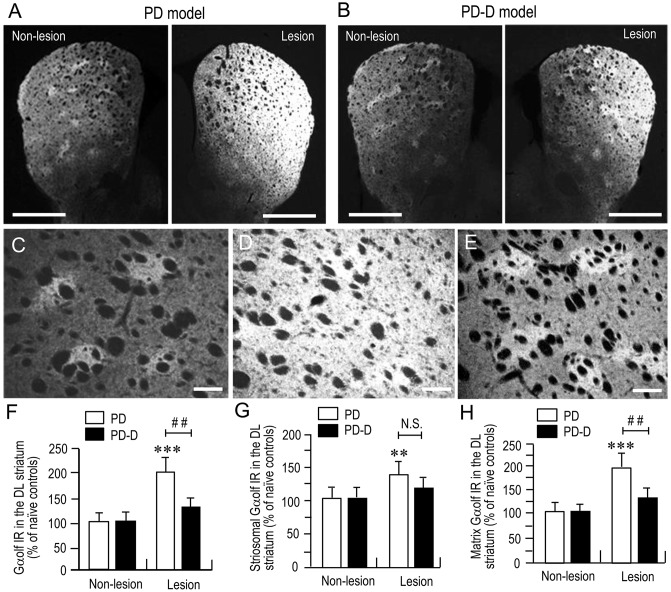
**Dopaminergic regulation of striatal Gα_olf_ levels. (A,B)** Representative low-magnification microscopic images of striatal sections stained for Gα_olf_ on the non-lesion and lesion sides from 6-OHDA-lesioned mice treated with daily injections of benserazide alone for 10 days (PD model; **(A)** and 6-OHDA-lesioned mice that received daily injections of benserazide and L-DOPA for 10 days and exhibited dyskinesia (PD-D model; **B**). **(C–E)** Representative higher-magnification microscopic images of the DL striatum stained for Gα_olf_ from naïve control **(C)**, PD **(D)** and PD-D **(E)** mice. **(F–H)** Optical density quantification of Gα_olf_ IR in the DL striatum on the non-lesion and lesion sides from PD (*n* = 15) and PD-D (*n* = 15) mice. Data are expressed as percentage of naïve control mice (*n* = 15) and are means ± SEM. **(F)** Quantification of Gα_olf_ IR in the DL regions in the striatum. ***P < 0.001 vs. naïve controls; ^##^*P* < 0.01 vs. PD; two-way analysis of variance (ANOVA) (*F*_(1,56)_ = 75.5) followed by Bonferroni’s test. **(G)** Quantification of Gα_olf_ IR in the striosome subfields in the DL striatum. **P < 0.01 vs. naïve controls; N.S. (not significant) vs. PD; two-way ANOVA (*F*_(1,56)_ = 9.2) followed by Bonferroni’s test. **(H)** Quantification of Gα_olf_ IR in the matrix subfields in the DL striatum. ***P < 0.001 vs. naïve controls; ^##^*P* < 0.01 vs. PD; two-way ANOVA (*F*_(1,56)_ = 89.6) followed by Bonferroni’s test. Scale bars: **(A,B)** = 2 mm; **(C–E)** = 100 μm.

To examine the dopaminergic regulation of Gα_olf_ protein levels in the striatum, we performed a quantitative IHC using an anti-Gα_olf_ antibody on striatal sections prepared from naïve control, PD, and PD-D mice. In low-magnification microscopic images, PD mice (Figure 3A) showed dramatic increases in Gα_olf_ IR in the dorsal striatum on the 6-OHDA-lesioned side when compared to non-lesioned side. In contrast, PD-D mice (Figure 3B) only had a modest increase in striatal Gα_olf_ IR on the 6-OHDA-lesioned side relative to non-lesioned side. Higher-magnification microscopic images of the DL striatum also show that, when compared to naïve controls (Figure 3C), there is a marked, but, slight increase in Gα_olf_ IR in the 6-OHDA-lesioned striatal areas of PD (Figure 3D) and PD-D (Figure 3E) mice. As indicated in a previous report (Ruiz-DeDiego et al., 2015), it is likely that dopamine depletion increases Gα_olf_ IR mainly in the matrix of the 6-OHDA-lesioned striatum, leading to a loss of the striosome-predominant pattern of Gα_olf_ IR expression in PD mice. However, daily treatment with L-DOPA reverses the lesion-induced increase in Gα_olf_ IR primarily in the matrix, leading to reappearance of the striosome-predominant pattern of Gα_olf_ IR expression in PD-D mice. These visual impressions were confirmed by quantitative densitometry analyses of the DL striatum (Figures 3F–H), as follows. We found a significant and marked increase of 101% (*P* < 0.001, two-way ANOVA) in Gα_olf_ IR levels in the 6-OHDA-lesioned striatum of PD, when compared to naïve controls. There was a 67% decrease (*P* < 0.01, two-way ANOVA) in Gα_olf_ IR levels in the 6-OHDA-lesioned striatum of PD-D mice when compared to that of PD mice (Figure 3F; PD mice: non-lesion, 102 ± 17% and 6-OHDA-lesion, 201 ± 28%; PD-D mice: non-lesion, 103 ± 19% and 6-OHDA-lesion, 134 ± 16%; % of naïve control mice ± SEM; *n* = 15).

In the striatal compartments of the DL striatum, we found a significant increase of 38% (*P* < 0.01, two-way ANOVA) Gα_olf_ IR levels in the striosomes of 6-OHDA-lesioned striatum of PD, when compared to naïve controls. There was no apparent difference (*P* > 0.05, two-way ANOVA) in striosomal levels of Gα_olf_ IR in the 6-OHDA-lesioned striatum between PD and PD-D mice (Figure 3G; PD mice: non-lesion, 101 ± 22% and 6-OHDA-lesion, 138 ± 26%; PD-D mice: non-lesion, 102 ± 19% and 6-OHDA-lesion, 122 ± 20%; % of naïve control mice ± SEM; *n* = 15). We also found a significant increase of 96% (*P* < 0.001, two-way ANOVA) in matrix levels of Gα_olf_ IR in the 6-OHDA-lesioned striatum of PD mice, when compared to those of naïve controls. There was a 66% decrease (*P* < 0.01, two-way ANOVA) in matrix levels of Gα_olf_ IR in the 6-OHDA-lesioned striatum of PD-D mice when compared to those of PD mice (Figure 3H; PD mice: non-lesion, 99 ± 23% and 6-OHDA-lesion, 196 ± 24%; PD-D mice: non-lesion, 102 ± 12% and 6-OHDA-lesion, 130 ± 22%; % of naïve control mice ± SEM; *n* = 15). These findings indicate that dopamine depletion causes a dramatic increase in Gα_olf_ levels in the DL striatum, particularly in the matrix. Daily exposure to L-DOPA induces a down-regulation of this lesion-induced increase in Gα_olf_ expression.

### Dopaminergic Regulation of Striatal Expression of D_1_R, A_2A_R, and D_2_R

**Figure 4:**
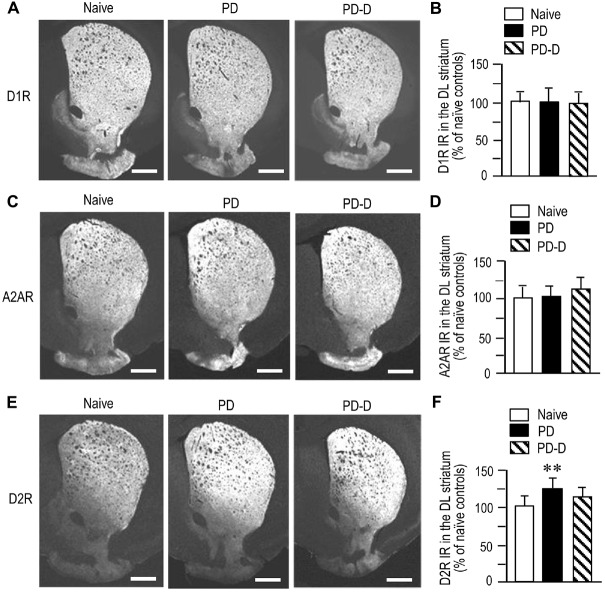
**Dopaminergic regulation of striatal expression of D_1_R, A_2A_R and D_2_R. (A)** Representative photomicrographs of striatal expression of D_1_R in normal (naïve controls) and lesioned hemispheres from 6-OHDA-lesioned mice treated with daily injections of benserazide alone for 10 days (PD model), and from 6-OHDA-lesioned mice that received daily injections of benserazide and L-DOPA for 10 days and exhibited dyskinesia (PD-D model). **(B)** Optical density quantification of D_1_R IR in the DL striatum from PD (*n* = 15) and PD-D (*n* = 15) mice. Data are expressed as percentage of naïve control mice (*n* = 15) and are means ± SEM. No significant changes in striatal levels of D_1_R IR in PD and PD-D mice were observed when compared to naïve controls; one-way ANOVA (*F*_(2,42)_ = 0.0) followed by Bonferroni’s test. **(C)** Representative photomicrographs of striatal expression of A_2A_R from naïve control, PD and PD-D mice. **(D)** Optical density quantification of A_2A_R IR in the DL striatum from PD (*n* = 15) and PD-D (*n* = 15) mice. Data are expressed as percentage of levels in naïve control mice (*n* = 15) and are means ± SEM. No significant changes in striatal levels of A_2A_R IR in PD and PD-D mice were observed when compared to naïve controls; one-way ANOVA (*F*_(2,42)_ = 1.2) followed by Bonferroni’s test. **(E)** Representative photomicrographs of striatal expression of D_2_R from naïve control, PD and PD-D mice. **(F)** Optical density quantification of D_2_R IR in the DL striatum from PD (*n* = 15) and PD-D (*n* = 15) mice. Data are expressed as percentage of levels in naïve control mice (*n* = 15) and are means ± SEM. **P < 0.01 vs. naïve controls; one-way ANOVA (*F*_(2,42)_ = 16.9) followed by Bonferroni’s test. Scale bars: **(A,C,E)** = 1 mm.

To examine dopaminergic regulation of D_1_R, A_2A_R and D_2_R expression in the DL striatum, we performed quantitative IHC on sections prepared from 6-OHDA-lesioned striata of PD and PD-D mice (Figure 4). We observed no significant changes (*P* > 0.05, one-way ANOVA) in the expression levels of D_1_R IR in PD or PD-D mice when compared to naïve controls (Figures 4A,B; PD mice, 101 ± 21%; PD-D mice, 98 ± 17%; % of naïve control mice ± SEM; *n* = 15). The expression levels of A_2A_R IR in PD and PD-D mice were not significantly different (*P* > 0.05, one-way ANOVA) from those in naïve controls (Figures 4C,D; PD mice, 103 ± 15%; PD-D mice, 118 ± 13%; % of naïve control mice ± SEM; *n* = 15). We found a significant increase in the expression of D_2_R in PD (*P* < 0.01, one-way ANOVA), but not PD-D (*P* > 0.05, one-way ANOVA), mice when compared to naïve controls (Figures 4E,F; PD mice, 123 ± 15%; PD-D mice, 112 ± 22%; % of naïve control mice ± SEM; *n* = 15). These findings indicate that dopamine depletion causes a significant increase in striatal D_2_R expression, which is reversed by daily treatment with L-DOPA. In addition, dopamine depletion and L-DOPA replacement cause no significant changes in striatal expression of D_1_R and A_2A_R in the dopamine-denervated striatum.

### Dopaminergic Regulation of Striatal Levels of PLA Signals for D_1_R-Gα_olf_

**Figure 5:**
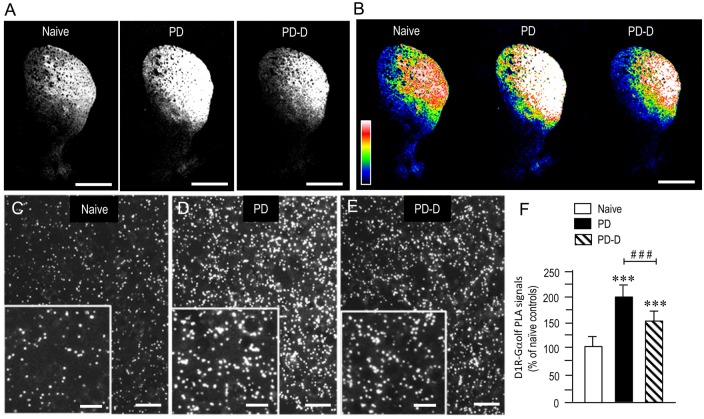
**Dopaminergic regulation of striatal levels of Gα_olf_ proteins in close proximity to D_1_R proteins.** Dual-antigen recognition *in situ* PLA used to detect Gα_olf_ proteins in proximity to D_1_R proteins (D_1_R-Gα_olf_) was carried out on normal hemispheres of naïve controls and on lesioned hemispheres from 6-OHDA-lesioned mice treated with daily injections of benserazide alone for 10 days (PD model) and from 6-OHDA-lesioned mice that received daily injections of benserazide and L-DOPA for 10 days and exhibited dyskinesia (PD-D model). **(A,B)** Representative photomicrographs of striatal expression of D_1_R-Gα_olf_ PLA signals in normal and lesioned hemispheres from PD and PD-D mice **(A)**, and their graded color-converted images **(B)**. **(C–E)** Representative photomicrographs of the DL striatum stained with the *in situ* PLA for D_1_R-Gα_olf_ from naïve control **(C)**, PD **(D)** and PD-D **(E)** mice. Their higher-magnification images are also shown in the *insets* in **(C–E)**. **(F)** Optical density quantification of D_1_R-Gα_olf_ PLA signals in the DL striatum from PD (*n* = 10) and PD-D (*n* = 10) mice. Data are expressed as percentage of naïve control mice (*n* = 10) and are means ± SEM. ***P < 0.001 vs. normal controls; ^###^*P* < 0.001 vs. PD; one-way ANOVA (*F*_(2,27)_ = 107.2) followed by Bonferroni’s test. Scale bars: **(A,B)** = 2 mm; **(C–E)** = 25 μm; *insets* in **(C–E)** = 10 μm.

To examine the dopaminergic regulation of striatal levels of Gα_olf_ protein in close proximity to D_1_R protein, we used a sensitive *in situ* PLA in sections prepared from 6-OHDA-lesioned striata from PD and PD-D mice (Figure 5). In low-magnification microscopic images, a marked and moderate increase in D_1_R-Gα_olf_ PLA signals was observed in the dorsal striatum in PD and PD-D mice when compared to naïve controls (Figures 5A,B). Higher-magnification microscopic images of the DL striatum also show that compared to naïve controls (Figure 5C), there is a marked and moderate increase in the D_1_R-Gα_olf_ PLA signals in 6-OHDA-lesioned striatal areas in PD (Figure 5D) and PD-D (Figure 5E) mice. Quantitative densitometry analyses of the DL striatum revealed increases of 92% (*P* < 0.001, one-way ANOVA) and 50% (*P* < 0.001, one-way ANOVA) in the D_1_R-Gα_olf_ PLA signal in PD and PD-D mice, respectively, when compared to naïve controls. There was a decrease of 42% (*P* < 0.001, one-way ANOVA) in the D_1_R-Gα_olf_ PLA signal in PD-D mice when compared to PD mice (Figure 5F; PD mice, 192 ± 25%; PD-D mice, 150 ± 21%; % of naïve control mice ± SEM; *n* = 10). These findings indicate that dopamine depletion causes a marked increase in striatal D_1_R-Gα_olf_ PLA signal, which is downregulated by daily treatment with L-DOPA. However, there is a significant increase of striatal D_1_R-Gα_olf_ PLA signal in PD-D mice compared to naïve controls.

### Dopaminergic Regulation of Striatal Levels of PLA Signals for A_2A_R-Gα_olf_

**Figure 6:**
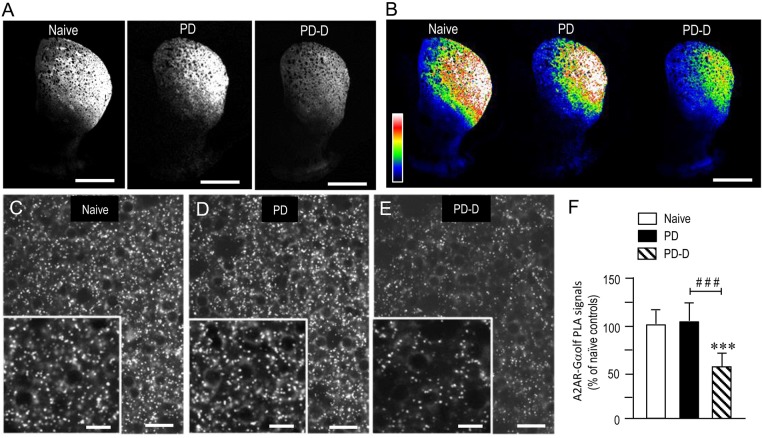
**Dopaminergic regulation of striatal levels of Gα_olf_ proteins in close proximity to A_2A_R proteins.** Dual-antigen recognition *in situ* PLA used to detect Gα_olf_ proteins in proximity to A_2A_R proteins (A_2A_R-Gα_olf_) was carried out on normal hemispheres of naïve controls and on lesioned hemispheres from 6-OHDA-lesioned mice treated with daily injections of benserazide alone for 10 days (PD model) and from 6-OHDA-lesioned mice that received daily injections of benserazide and L-DOPA for 10 days and exhibited dyskinesia (PD-D model). **(A,B)** Representative photomicrographs of striatal expression of A_2A_R-Gα_olf_ PLA signals in normal and lesioned hemispheres from PD and PD-D mice **(A)**, and their graded color-converted images **(B)**. **(C–E)** Representative photomicrographs of the DL striatum stained with the *in situ* PLA for A_2A_R-Gα_olf_ from naïve control **(C)**, PD **(D)** and PD-D **(E)** mice. Their higher-magnification images are also shown in the *insets* in **(C–E)**. **(F)** Optical density quantification of A_2A_R-Gα_olf_ PLA signals in the DL striatum from PD (*n* = 10) and PD-D (*n* = 10) mice. Data are expressed as percentage of naïve control mice (*n* = 10) and are means ± SEM. ***P < 0.001 vs. naive controls; ^###^*P* < 0.001 vs. PD; one-way ANOVA (*F*_(2,27)_ = 72.6) followed by Bonferroni’s test. Scale bars: **(A,B)** = 2 mm; **(C–E)** = 25 μm; *insets* in **(C–E)** = 10 μm.

To examine the dopaminergic regulation of striatal levels of Gα_olf_ protein in close proximity to A_2A_R protein, we used a sensitive *in situ* PLA in sections prepared from 6-OHDA-lesioned striata from PD and PD-D mice (Figure 6). Notably, low-magnification microscopic images show an apparent decrease in the A_2A_R-Gα_olf_ PLA signal in the DL striatum of PD-D mice when compared to both naïve control and PD mice (Figures 6A,B). Higher-magnification images also show the localization patterns of A_2A_R-Gα_olf_ PLA signals in the DL striatum of naïve control (Figure 6C), PD (Figure 6D), and PD-D (Figure 6E) mice. Quantitative densitometry analyses of the DL striatum revealed decreases of 41% (*P* < 0.001, one-way ANOVA) and 45% (*P* < 0.001, one-way ANOVA) in A_2A_R-Gα_olf_ PLA signal levels in PD-D mice, when compared to naïve controls and PD mice, respectively (Figure 6F; PD mice, 104 ± 24%; PD-D mice, 59 ± 21%; % of naïve control mice ± SEM; *n* = 10). These findings indicate that L-DOPA replacement, but not dopamine depletion, causes a significant decrease in the striatal A_2A_R-Gα_olf_ PLA signal in the dopamine-denervated striatum.

### Differences in Striatal Responsiveness to Dopamine Stimulation between PD and PD-D Mice

**Figure 7:**
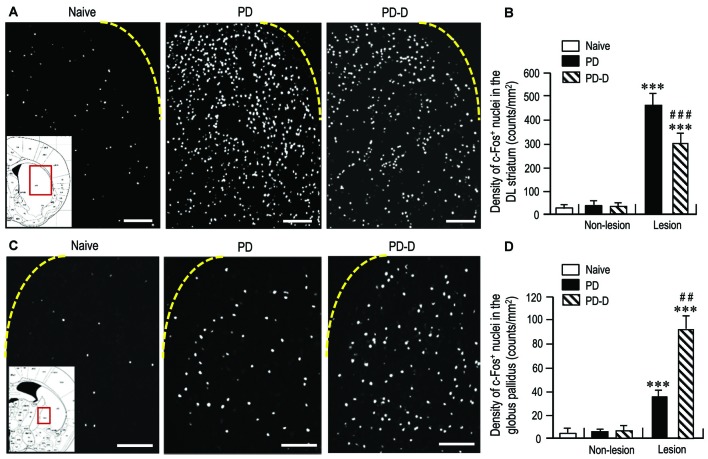
**Effects of dopamine stimulation on striatal and pallidal c-Fos expression in 6-OHDA-lesioned mice.** An immunohistochemical study with anti-c-Fos antibody was carried out on the striatal sections from PD and PD-D mice, which received injections of L-DOPA (20 mg/kg) and benserazide-HCl (12 mg/kg) 2 h before sacrifice on day 11 (see Figure 1A), and those from naïve controls that also received the injections of L-DOPA (20 mg/kg) and benserazide-HCl (12 mg/kg) 2 h before sacrifice. The density measurements were performed by counting the numbers of c-Fos-positive (c-Fos^+^) nuclei in a 0.5 mm × 0.5 mm field in the striatum and globus pallidus from each animal. **(A)** Representative photomicrographs of the DL striatum stained for c-Fos in naïve control, PD and PD-D mice. The *inset* (red open box) in the naïve control is the corresponding figure from the atlas of Paxinos and Franklin (2001) to show the striatal area that were analyzed in all naïve control, PD and PD-D mice. **(B)** Density quantification of c-Fos^+^ nuclei in the DL striatum from naïve controls (*n* = 10), and of those on non-lesion and lesion sides in PD (*n* = 10) and PD-D (*n* = 10) mice. Data are expressed as means ± SEM. ***P < 0.001 vs. naïve controls; ^###^*P* < 0.001 vs. PD; two-way ANOVA (*F*_(1,36)_ = 140.9) followed by Bonferroni’s test. **(C)** Representative photomicrographs of the globus pallidus stained for c-Fos in naïve control, PD and PD-D mice. The *inset* (red open box) in the naïve control is the corresponding figure from the atlas of Paxinos and Franklin (2001) to show the pallidal area that were analyzed in all naïve control, PD and PD-D mice. **(D)** Density quantification of c-Fos^+^ nuclei in the globus pallidus from naïve controls (*n* = 10), and of those on non-lesion and lesion sides in PD (*n* = 10) and PD-D (*n* = 10) mice. Data are expressed as means ± SEM. ***P < 0.001 vs. naïve controls; ^##^*P* < 0.01 vs. PD; two-way ANOVA (*F*_(1,36)_ = 80.8) followed by Bonferroni’s test. Scale bars: **(A)** = 200 μm; **(C)** = 100 μm.

To assess changes in striatal responsiveness to dopamine stimulation in PD and PD-D mice, we performed IHC using an antibody against c-Fos, which is known to be induced in the striatum and globus pallidus following the stimulation of D_1_Rs and D_2_Rs (Marshall et al., 1993; LaHoste and Marshall, 1994). We prepared striatal sections from PD and PD-D mice that received injections of L-DOPA (20 mg/kg) and benserazide-HCl (12 mg/kg) 2 h before sacrifice on day 11 (see Figure 1A). Microscopic images of the DL striatum stained for c-Fos from naïve control, PD and PD-D mice are shown in Figure 7A. Compared to naïve controls that also received the injections of L-DOPA (20 mg/kg) and benserazide-HCl (12 mg/kg) 2 h before sacrifice, we found a marked increase in the densities of c-Fos-positive (c-Fos^+^) nuclei in the 6-OHDA-lesioned striatum in both PD and PD-D mice. Quantitative densitometry analyses also showed a marked increase (*P* < 0.001, two-way ANOVA) in the density of c-Fos^+^ nuclei in the 6-OHDA-lesioned striatum in both PD and PD-D mice when compared to naïve controls. However, there was a decrease of ~40% (*P* < 0.001, two-way ANOVA) in the density of c-Fos^+^ nuclei in the 6-OHDA-lesioned striatum of PD-D mice when compared to PD mice (Figure 7B; naïve controls: 20 ± 12; PD mice: non-lesion, 31 ± 10 and 6-OHDA-lesion, 475 ± 55; PD-D mice: non-lesion, 29 ± 12 and 6-OHDA-lesion, 292 ± 49; means ± SEM; *n* = 10). Microscopic images of the globus pallidus stained for c-Fos obtained from naïve control, PD, and PD-D mice are shown in Figure 7C. Compared to naïve controls, we found increased densities of c-Fos^+^ nuclei in the globus pallidus on the lesioned sides in both PD and PD-D mice. Quantitative densitometry analyses also indicated a significant increase (*P* < 0.001, two-way ANOVA) in the density of c-Fos^+^ nuclei in the globus pallidus on the lesioned sides in both PD and PD-D mice when compared to naïve controls. Importantly, we found that there was an increase of ~140% (*P* < 0.01, two-way ANOVA) in the density of c-Fos^+^ nuclei in the globus pallidus on the lesioned side in PD-D mice when compared to PD mice (Figure 7D; naïve controls: 4 ± 2; PD mice: non-lesion, 5 ± 3 and 6-OHDA-lesion, 38 ± 8; PD-D mice: non-lesion, 8 ± 7 and 6-OHDA-lesion, 92 ± 11; means ± SEM; *n* = 10). These findings indicate that dopamine depletion causes a marked increase in the responsiveness of striatal D1-cells to dopamine stimulation, which is downregulated by daily treatments with L-DOPA. Given the changes in striatal D_2_R expression in PD and PD-D mice (see above), it is likely that in the dopamine-denervated striatum, dopamine depletion may cause increased striatal D_2_R expression, which then enhances the responsiveness of D2-cells to dopamine stimulation (Cai et al., 2002). Notably, L-DOPA replacement could induce a further increase in the responsiveness of D2-cells to dopamine stimulation despite no obvious increase in striatal D_2_R expression in the dopamine-denervated striatum.

## Discussion

**Figure 8:**
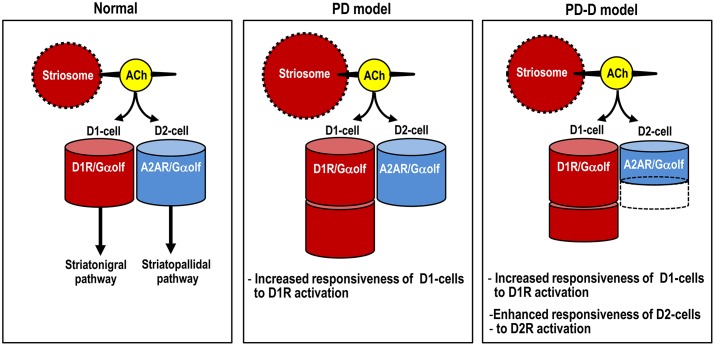
**Proposed diagram for dopaminergic regulation of Gα_olf_ levels that determine responsiveness to dopamine stimulation in striatal D1- and D2-cells.** The heights of the red and blue columns indicate the abundance of Gα_olf_ proteins in D1-cells and D2-cells, respectively. In PD mice, D1-cells might exhibit the dopamine D_1_R hypersensitivity caused by a dramatic increase in their Gα_olf_ levels, while D2-cells might show no apparent changes in their Gα_olf_ levels. In PD-D mice, D1-cells might show an increase in their Gα_olf_ levels, while D2-cells might show a decrease in their Gα_olf_ levels, which might result in an enhanced responsiveness to D_2_R activation. Abbreviations: PD, Parkinson’s disease: PD-D, PD with dyskinesia; ACh, acetylcholine; D1-cell, dopamine D1 receptor-expressing striatonigral medium spiny neuron; D2-cell, dopamine D2 receptor-expressing striatopallidal medium spiny neuron; D1R, dopamine D_1_ receptor; D2R, dopamine D_2_ receptor; Gαolf, olfactory type G-protein α subunit.

Here we used IHC and *in situ* PLA to determine the region- and cell-type- specific distributions of Gα_olf_ proteins in the mouse striatum. Using a mouse model of hemiparkinsonism induced by 6-OHDA, we also found that daily *pulsatile* administration of L-DOPA might induce usage-dependent changes in Gα_olf_ expression not only in D1-cells, but also in D2-cells in the dopamine-depleted striatum (see Figure 8). This raises the possibility that LID might result from reduced A_2A_R/Gα_olf_/cAMP signal levels in D2-cells, which may be caused by intermittent and *pulsatile* activation of postsynaptic D_1_Rs in the striatum. Our results support and provide new insights into the hypothesis that LID is associated with a decrease in activity of “indirect” striatopallidal pathway (Crossman, 1990; DeLong, 1990; Brotchie, 2005; Guridi et al., 2012).

### Strategic Localization of Gα_**olf**_ Proteins in the Striatum

We used IHC to demonstrate that Gα_olf_ IR is highly concentrated in the DL striatum, which corresponds to the motor-sensory territory in rodents and is analogous to the putamen in primates (Graybiel, 2008). This implies that Gα_olf_ may have a unique position in regulating the activities of the cortico-thalamo-basal ganglia circuit involved in motor functions, i.e., the motor loop (Alexander and Crutcher, 1990), at the striatal level. Although a previous study revealed no obvious compartmental difference in Gα_olf_ mRNA expression throughout striatal development in rats (Sakagami et al., 1995), we observed differential concentrations of Gα_olf_ IR in the striosome and matrix compartments, with higher densities of Gα_olf_ IR in the striosomes relative to the matrix. This finding suggests that Gα_olf_ may be a key molecule for controlling differential responses of striosome-matrix systems to D_1_R activation in adult mice. There is evidence that in experimental animal models with 6-OHDA-lesions (Hervé et al., 1993; Corvol et al., 2004; Alcacer et al., 2012; Ruiz-DeDiego et al., 2015) or in those with a total absence of D_1_Rs due to *D_1_R* gene targeting (Hervé et al., 2001), the upregulation of Gα_olf_ levels in the striatum is not accompanied by a parallel increase in Gα_olf_ mRNA expression. Thus, homeostatic regulation of striatal Gα_olf_ protein levels is thought to occur via post-translational mechanisms, wherein the altered expression of Gα_olf_ protein depends directly on its rate of usage (Hervé, 2011). We suggest that when compared to the matrix, the striosomes might have the lower levels of D_1_R/Gα_olf_ stimulation, which may then lower the Gα_olf_ degradation rate and lead to accumulation of the protein. Our assumption is supported by the present finding that changes in striatal Gα_olf_ IR expression were primarily found in the matrix in both PD and PD-D mice.

In this study, we first used highly sensitive dual-antigen recognition *in situ* PLA using a combination of the Brightfield Duolink PLA kit reagents and the TSA system (see “Materials and Methods” Section). This dual-antigen recognition PLA technique allowed us to obtain specific and efficient fluorescent signals showing Gα_olf_ protein in close proximity to D_1_R or A_2A_R protein in the striatum. However, we cannot say that all the PLA signals detected here resulted from the direct interaction (or actual coupling) of Gα_olf_ protein with D_1_R or A_2A_R protein. Borroto-Escuela et al. (2013) have shown that PLA can indicate a close proximity between two proteins, which is not always a reflection of direct interaction. This is because *in situ* PLA signals can be detected when two protein epitopes are in close proximity with ranges of 10–30 nm or more. In addition, although the precise mechanisms by which Gα_olf_ protein interact with D_1_R or A_2A_R protein remains unclear, it was also noted that in striatal membrane, the content in Gα_olf_ protein would be almost one to two orders of magnitude higher than that in D_1_R or A_2A_R (Hervé, 2011).

### Striatal Gα_olf_ as a Determinant of the Increased Responsiveness of D1-cells to Dopamine Stimulation in LID

As shown in previous studies (Alcacer et al., 2012; Ruiz-DeDiego et al., 2015), we found a marked increase in striatal Gα_olf_ protein levels in PD mice with 6-OHDA lesions. This is in line with evidence that dopamine depletion may lead to up-regulation of Gα_olf_ protein expression in the rat striatum (Hervé et al., 1993; Marcotte et al., 1994; Penit-Soria et al., 1997; Corvol et al., 2004; Rangel-Barajas et al., 2011) and in the putamen in patients with PD (Corvol et al., 2004). Given the evidence that striatal levels of D_1_R (Shinotoh et al., 1993; Turjanski et al., 1997: Hurley et al., 2001) and other major mediators of D_1_R signaling (Girault et al., 1989; Nishino et al., 1993) are unchanged in PD patients, the dramatic increase in striatal Gα_olf_ protein level may be a key event in the D_1_R hypersensitivity that develops in PD (Alcacer et al., 2012). In support of this notion, we detected no obvious changes in striatal D_1_R expression in PD mice.

Previous data have suggested that the up-regulation of Gα_olf_ protein levels in the dopamine-depleted striatum is post-translational (Hervé et al., 1993; Ruiz-DeDiego et al., 2015) and results from the disuse of the D_1_Rs (Hervé et al., 2001). Indeed, daily administration of L-DOPA for 10 days resulted in a down-regulation of the increased Gα_olf_ protein levels in the 6-OHDA-lesioned striatum in PD-D mice. However, we also found a significant increase in striatal Gα_olf_ levels in PD-D mice when compared to naïve controls. In agreement with the changes in striatal Gα_olf_ levels in PD and PD-D mice, *in situ* PLA also revealed that striatal D_1_R-Gα_olf_ PLA signals were dramatically increased in PD mice and moderately increased in PD-D mice. These findings imply an increased responsiveness of D1-cells to D_1_R activation in PD-D mice, although this responsiveness is lower than that found in PD mice. Our assumption is also supported by the fact that, compared to naïve controls, a significant increase in the number of striatal c-Fos^+^ nuclei consequent to L-DOPA administration was evident in PD-D mice, although this increase was more pronounced in PD mice. Since Gα_olf_ represents the rate-limiting factor in D_1_R-mediated cAMP production in D1-cells, these findings suggest that striatal Gα_olf_ level acts as a determinant for the increased responsiveness of D1-cells to dopamine stimulation in LID (see Figure 8).

### Striatal Gα_**olf**_ as a Determinant of the Increased Responsiveness of D2-cells to Dopamine Stimulation in LID

It has been postulated that repeated exposure to dopaminergic agents leads to increased sensitivity of D2-cells to D_2_R activation in the dopamine-depleted striatum in experimental animals (Engber et al., 1989; Asin et al., 1995; Kashihara et al., 2000). However, no obvious increase in striatal D_2_R expression has been observed in PD patients treated with dopaminergic drugs (Rinne et al., 1981; Guttman and Seeman, 1985; Antonini et al., 1997; Thobois et al., 2004). In agreement with this notion, we found that pallidal c-Fos induction consequent to L-DOPA administration was more marked in PD-D mice compared to PD mice. On the other hand, there was increased expression of striatal D_2_Rs in PD mice, but not in PD-D mice. This indicates that the repeated administration of L-DOPA results in an increased responsiveness of D2-cells to striatal D_2_R activation in the dopamine-denervated striatum, and suggests that this phenomenon might underlie LID. Using an *in situ* PLA, we found that A_2A_R-Gα_olf_ PLA signals were markedly reduced along with Gα_olf_ protein levels in the 6-OHDA-lesioned striatum of PD-D mice. This novel finding indicates that as in D1-cells, repeated exposure to L-DOPA causes down-regulation of Gα_olf_ protein levels in D2-cells in the dopamine-depleted striatum. This then leads to the facilitation of the effects of dopamine on D2-cells by reducing A_2A_R/Gα_olf_ signaling-mediated cAMP production (see Figure 8). This may be the reason that PD-D mice display an increased responsiveness of D2-cells to dopamine stimulation. However, the mechanism by which *repeated* and *pulsatile* injections of L-DOPA causes a decrease in A_2A_R/Gα_olf_ PLA signals in PD-D remains a matter of speculation, as follows.

A_2A_R usage by endogenous adenosine results in a basal rate of Gα_olf_ degradation (Hervé et al., 2001). It has been shown that in experimental animals with 6-OHDA-lesions, chronic (or persistent) dopamine depletion caused no significant changes (Ballarin et al., 1987; Herrera-Marschitz et al., 1994; Nomoto et al., 2000) or slight decrease (Pinna et al., 2002) in the extracellular adenosine levels in the striatum. In accordance with these findings, our present results also showed no significant changes in striatal levels of A_2A_R-Gα_olf_ PLA signals in PD mice. Thus, we suggest that *chronic* dopamine depletion *per se* might cause no obvious changes in A_2A_R/Gα_olf_ signaling activities that depend on the endogenous adenosine levels in striatal D2-cells. However, it is known that endogenous levels of adenosine are increased in response to the activation of *N*-methyl-D-aspartate (NMDA) receptors (Delaney and Geiger, 1998; Delaney et al., 1998), which can be facilitated by D_1_R stimulation (Cepeda and Levine, 2012; Morigaki and Goto, 2015), in the striatum. A landmark report has shown that in the rat striatum, *transient (pulsatile)* stimulation of D_1_Rs facilitates the NMDA receptor-dependent increase in extracellular adenosine levels (Harvey and Lacey, 1997). These findings suggest that in 6-OHDA-lesioned mice with D_1_R hypersensitivity, repeated exposure to L-DOPA may lead to a *transient* activation of D_1_Rs, which then enhances the NMDA receptor-dependent increase in adenosine release in the dopamine-denervated striatum. Moreover, Nash and Brotchie (2000) have shown that in striatal slices prepared from rats with 6-OHDA lesions, NMDA receptor activation could cause a marked increase in adenosine release and, thereby, indirectly stimulate A_2A_Rs. Taken together, we speculate that in the 6-OHDA-lesioned striatum of PD-D mice, decreased Gα_olf_ levels in D2-cells might be due to increased extracellular adenosine levels caused by the daily *pulsatile* activation of striatal D_1_Rs. If our assumption is correct, striatal D_1_R signals might contribute to regulation of the Gα_olf_ protein levels in not only D1-cells but also D2-cells in the dopamine-depleted striatum.

Because adenosine/A_2A_R signaling functionally opposes the actions of D_2_Rs on D2-cells by its ability to increase the A_2A_R/Gα_olf_-dependent cAMP production, it has so far been suggested that A_2A_R antagonism may boost the anti-parkinsonian action of D_2_R agonists in treating PD symptoms (Jenner, 2003; Schwarzschild et al., 2006; Fuxe et al., 2007; Huot et al., 2013). In addition, based on the evidence that striatal A_2A_R expression might be increased in PD patients with dyskinesia (Calon et al., 2004; Ramlackhansingh et al., 2011) and in dyskinetic animal models of PD (Jenner et al., 2009), it has also been suggested that adenosine A_2A_ sites might be a potential pharmacologic target for reducing LIDs (Jenner et al., 2009; Ramlackhansingh et al., 2011; Huot et al., 2013; Kanda and Uchida, 2014). In Japan, istradefylline, an A_2A_R antagonist, is currently used in clinics for treating PD patients (Kondo and Mizuno, 2015). The drug has shown to improve “off” time in patients with advanced PD, but has not shown anti-LID effects in the absence of a reduction in dopaminergic drug dosage. Adjunct use of istradefylline often causes dyskinetic symptoms as a major adverse effect (Kondo and Mizuno, 2015). Considering usage-dependent Gα_olf_ degradation through adenosine/A_2A_R, we assume that in PD patients treated with L-DOPA, adenosine/A_2A_R antagonism might be effective in reducing the “priming” of LID. However, once LID is established, adenosine/A_2A_R antagonism might exacerbate dyskinetic symptoms. Our assumption may corroborate the notion that A_2A_R activation might be required for dyskinesia “priming” mechanism (Brotchie, 2005).

## Conclusion

Because Gα_olf_ protein level serves as a determinant of cAMP signal-dependent activity in both D1-cells and D2-cells in the striatum, Gα_olf_ may represent an ideal target for the modulation of striatal functions under physiological and pathological conditions. Dysregulation of Gα_olf_ expression has been associated with the pathophysiology of several brain disorders (Hervé, 2011). Of our particular interest is that the *GNAL* gene, which encodes Gα_olf_, is a causative gene in primary (torsion) dystonia (Fuchs et al., 2013). This is direct evidence that Gα_olf_ plays a pivotal role in the “motor loop” of the cortico-basal ganglia circuits. Under parkinsonian conditions, dopamine depletion results in a crucial D_1_R hypersensitivity in the striatum, which leads to the beneficial effects of L-DOPA in PD patients, but also generates LID. In this study, we found that in the 6-OHDA-lesioned striatum of PD mice, daily pulsatile administrations of L-DOPA may cause usage-induced changes in striatal Gα_olf_ levels, leading to increased responsiveness to dopamine stimulation in both D1-cells and D2-cells. Thus we suggest that L-DOPA-induced changes in Gα_olf_ levels in the dopamine-depleted striatum may be a key event in LID development.

## Author Contributions

SG conceived and designed the experiments. RM, SO and SG performed the experiments; analyzed the data; contributed reagents/materials/analysis tools. SG wrote the manuscript.

## Conflict of Interest Statement

The authors declare that the research was conducted in the absence of any commercial or financial relationships that could be construed as a potential conflict of interest.
